# Inflammation and Hemoglobin Oxygen Affinity

**DOI:** 10.1007/s10753-025-02386-2

**Published:** 2025-12-18

**Authors:** Nikolai Staier, Norbert Mair, Christoph Frisch, Herbert Oberacher, Alexander Egger, Thomas Haller, Christopher Rugg, Simon Woyke

**Affiliations:** 1 Department of Anaesthesiology and Intensive Care Medicine, Medical University of Innsbruck, Anichstraße 35, 6020 Innsbruck, Austria; 2 Department of Physiology and Medical Physics, Institute of Physiology, Medical University of Innsbruck, Innsbruck, Austria; 3 Institute of Legal Medicine and Core Facility Metabolomics, Medical University of Innsbruck, Innsbruck, Austria; 4 Central Institute of Medical and Chemical Laboratory Diagnostics (ZIMCL), University Hospital of Innsbruck, Innsbruck, Austria

**Keywords:** Oxygen dissociation curve, Sepsis, Inflammation, Dexamethasone, Hemoglobin

## Abstract

In septic patients, oxygen delivery is often impaired, yet the specific impact of inflammation on erythrocyte oxygen transport characteristics remains largely unexplored. This study examined the direct effects of endotoxin-induced inflammation on the oxygen dissociation curve (ODC) and evaluated dexamethasone and noradrenaline as potential pharmacological interventions to modulate hemoglobin oxygen-binding properties during inflammation. Blood samples from ten healthy male volunteers were allocated to four groups: control, lipopolysaccharide (LPS), LPS with dexamethasone, and LPS with noradrenaline. Samples were incubated at 37 °C for two hours. ODCs were determined, providing p50 values and Hill coefficients. Blood gas analysis, 2,3-bisphosphoglycerate levels, and interleukin-6 (IL-6) levels were measured to assess metabolic parameters and inflammatory response. p50 values and Hill coefficients showed no significant differences between LPS-stimulated samples and controls. LPS effectively induced inflammatory activation with significantly elevated IL-6 levels (1416 (1226–1778) vs. 3 [2–4] ng/l, *p* = 0.002). Dexamethasone co-treatment significantly increased p50 values compared to LPS alone (29.2 (28.1–29.7) vs. 26.9 (25.7–27.9) mmHg, *p* = 0.030) and altered Hill coefficients (2.47 (2.32–2.56) vs. 2.65 (2.57–2.86), *p* = 0.002), indicating a rightward shift of the ODC. Noradrenaline demonstrated anti-inflammatory effects with reduced IL-6 levels (1212 (1068–1427) vs. 1416 (1226–1778) ng/l, *p* = 0.013) but did not significantly alter oxygen-binding properties. Acute inflammation does not directly alter hemoglobin oxygen-binding properties. The novel dexamethasone-induced rightward shift of the ODC may facilitate oxygen release to tissues and could have important clinical implications for steroid therapy in septic patients.

## Introduction

In critically ill and septic patients, oxygen delivery is frequently compromised due to reduced cardiac output, decreased arterial oxygen content, and microcirculatory dysfunction, potentially leading to severe tissue hypoxia and organ dysfunction [1]. While microvascular dysregulation in sepsis is well documented, the specific impact of inflammation on oxygen transport characteristics of red blood cells remains largely unexplored [2]. This knowledge gap is particularly relevant since oxygen supply-demand mismatch is a hallmark of septic shock and a major determinant of patient outcomes [3].

The oxygen dissociation curve (ODC), which describes the relationship between partial pressure of oxygen (pO_2_) and hemoglobin saturation (SO_2_), is fundamental to understanding oxygen transport and delivery to tissues. The hemoglobin-oxygen affinity, typically quantified by the p50 (the oxygen tension at which hemoglobin is 50% saturated), is influenced by various physiological factors. The Hill coefficient describes the cooperativity of oxygen binding to hemoglobin, reflecting the sigmoid shape of the ODC, indicating positive cooperativity where oxygen binding to one subunit facilitates binding to others. While the primary modulators include pH, temperature, partial pressure of carbon dioxide (pCO_2_), and 2,3-bisphosphoglycerate (2,3-BPG) concentration, other factors such as lactate levels, sex, and various pharmacologic agents can also affect this relationship [4, 5].

In the context of endotoxemia-induced inflammation, factors that modulate the ODC undergo complex changes. While septic shock frequently leads to lactic acidosis and inflammation can induce hyperthermia, the specific impact of these sepsis-associated physiological alterations on hemoglobin-oxygen binding properties remains incompletely characterized, despite the well-established effects of pH and temperature on the ODC under controlled conditions [4, 6, 7].

Recent evidence suggests that erythrocytes are not merely passive oxygen carriers but also active participants in inflammatory responses [8]. They can bind cytokines through the Duffy-antigen receptor for chemokines (DARC) and serve as dynamic cytokine reservoirs [9, 10]. However, how this inflammatory interaction affects their primary function, namely oxygen transport, remains poorly understood.

This study aims to examine the direct effects of inflammation on the ODC in vitro. Understanding these alterations is crucial for elucidating the pathophysiological mechanisms of oxygen transport dysfunction in endotoxemia and may provide insights into potential therapeutic targets for improving tissue oxygenation in septic patients.

## Methods

### Ethics Approval and Consent

This experimental ex-vivo study was conducted following approval from the Ethics Committee of the Medical University of Innsbruck (vote nr. 1166/2024). Written informed consent was obtained from all participants prior to study enrollment.

### Participants

Ten healthy male volunteers aged 18–40 years were recruited. Given the pilot nature of this investigation and known sex-dependent variations in the ODC, we initially restricted enrollment to male participants to establish baseline effect sizes. A subsequent study is planned to investigate potential sex-specific variations in the observed outcomes.

### Exclusion Criteria

Subjects were excluded if they were current or recent smokers, used anti-inflammatory medications, had recent exposure to high altitude, experienced recent major blood loss or surgery, had a history of hemoglobinopathy or hematological disease.

### Sample Collection

Venous blood samples (12 ml) were collected via peripheral venipuncture into heparinized tubes. Samples were stored on ice and analyzed within eight hours of collection.

### Experimental Groups and Interventions

**Fig. 1:**
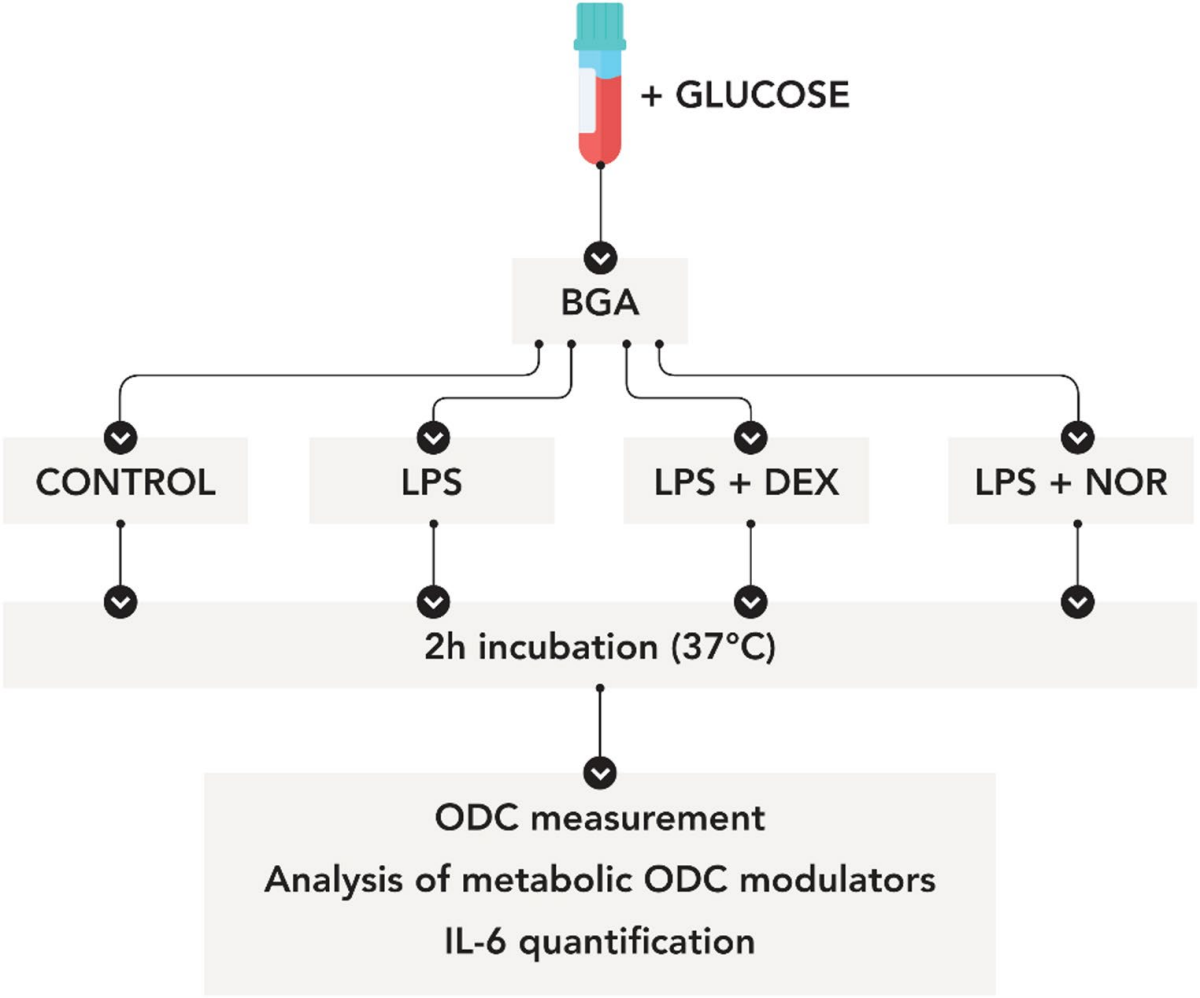
Experimental design for assessing the impact of LPS-induced inflammation and pharmacological interventions (dexamethasone and noradrenaline) on the oxygen dissociation curve (ODC)

Baseline blood gas analysis was performed immediately. To ensure adequate metabolic substrate availability during incubation, glucose was added to all samples (10 µl of 10% glucose solution per ml whole blood). Each sample was divided into four aliquots (V = 3 ml) to investigate distinct aspects of inflammatory modulation (see Fig. 1). The control group samples remained unstimulated to establish baseline values. The second set of samples received lipopolysaccharide (LPS) alone to characterize the direct inflammatory response. To examine pharmacological modulation of inflammation, the third set of samples was treated with both dexamethasone and LPS to assess anti-inflammatory effects, while the fourth set of samples received noradrenaline with LPS to evaluate noradrenaline-induced immunomodulation.

To induce a maximal inflammatory response within the short incubation period, we used LPS from Salmonella enteritidis (L7770, Sigma-Aldrich) at a final concentration of 1.0 µg/ml in whole blood [11]. Dexamethasone (D2915, water-soluble, Sigma-Aldrich) was added to yield a final concentration of 1 µM, sufficient to exert maximal anti-inflammatory effects [12]. Noradrenaline was administered at 0.2 µg/ml, a dose shown to produce significant immunomodulatory effects in previous studies [13].

All samples were incubated at 37 °C for two hours in closed monovettes with constant gentle rotation to prevent clotting and sedimentation. This incubation period was selected to ensure sufficient time for inflammatory response development while preventing significant ex vivo erythrocyte deterioration. Following incubation, blood gas analysis was performed, and aliquots of the samples were centrifuged (1500 × g for 10 min at 4 °C) to separate plasma. The inflammatory response was quantified by measuring interleukin-6 (IL-6) levels in plasma. IL-6 was determined using an electrochemiluminescence immunoassay on a Cobas 8000 analyzer (Roche Diagnostics, Rotkreuz, Switzerland). The standardized hemolysis index (a measure of free hemoglobin in plasma indicating red blood cell lysis) was used to estimate the degree of hemolysis in the samples.

### ODC Determination

ODCs were determined using a previously described high-throughput method [14]. Briefly, blood samples were prepared as thin blood films to facilitate rapid gas exchange, while oxygen concentration in the gaseous phase was gradually reduced from 20 vol % to 0 vol % over a 20-minute period. A spectrophotometric plate reader measured pO₂ and SO₂ simultaneously at one-minute intervals, generating sufficient data points for accurate curve fitting. For quality control and validation, we used an internal hemoglobin standard to verify measurement consistency across experiments and a positive control with 5-hydroxymethylfurfural (5-HMF), a well-established agent that induces a leftward shift of the ODC [15]. All materials, including gas mixtures and blood films, were pre-warmed to 37 °C, and pCO₂ was maintained at 40 mmHg throughout measurements to standardize the Bohr effect.

### 2,3-BPG and ATP Quantification

Aliquots of the collected blood samples (30 µl) were stored at -80 °C for the analysis of metabolic ODC modulators. Concentrations of 2,3-BPG and ATP were determined with liquid chromatography-tandem mass spectrometry. The method enables the reliable quantitative analyses of the two targets in the range 50 − 10,000 µg/ml blood. Isotopically labeled analogues (ATP-^13^C_10_ and 2,3-BPG-^13^C_3_) were used as internal standards. Sample processing of blood samples (10 µl) involved protein precipitation with methanol and dilution [16].

### Statistical Analysis

Statistical analysis was performed using R (v4.4.2, R Core Team, www.R-project.org) and RStudio (2024.12.0 + 467, RStudio Inc., Boston, MA). Data normality was assessed using the Shapiro-Wilk test. Due to non-normal distribution of the data, non-parametric statistical tests were applied. Paired comparisons between experimental groups and controls were performed using the Wilcoxon signed-rank test. Data are presented as median with interquartile range (Q1-Q3). A p-value < 0.05 was considered statistically significant.

## Results

Mean age of the healthy male participants was 33 years. Table 1 shows the measured key indicators of the oxygen dissociation curve.

**Table 1:** Oxygen dissociation curve parameters across experimental groups after incubation. Values are presented as median (Q1-Q3), *n* = 10. P-values represent comparison with the unstimulated control group using a paired Wilcoxon-Rank sum test. Significant p-values (*p* < 0.05) are marked in bold

Parameter	Control	LPS	LPS + Dexamethasone	LPS + Noradrenaline
p50 (mmHg)	26.9 (26.4–28.4)	26.9 (25.7–27.9; *p* = 0.49	29.2 (28.1–29.7); *p* = 0.02	27.3 (26.7–27.8); *p* = 0.68
Hill coefficient	2.63 (2.53–2.81)	2.65 (2.57–2.86); *p* = 0.43	2.47 (2.32–2.56); *p* < 0.001	2.66 (2.58–2.79); *p* = 0.56
Metabolic Modulators				
2,3-BPG (µmol/gHb)	10.9 (9.7–11.0)	10.1 (9.5–10.5); *p* = 0.19	9.6 (8.7–10.5); *p* = 0.08	-
ATP (mmol/l)	2.57 (2.39–2.96)	2.55 (2.37–2.88); *p* = 0.28	2.56 (2.37–2.85); *p* = 0.13	-
Blood Gas Parameters				
pH	7.39 (7.34–7.40)	7.38 (7.35–7.42); *p* = 0.19	7.40 (7.35–7.41); *p* = 0.57	7.39 (7.37–7.42); *p* = 0.15
pCO₂ (mmHg)	34 (31–38)	32 (30–34); *p* = 0.02	31 (29–34); *p* = 0.07	30 (27–34); *p* = 0.009
Lactate (mmol/l)	4.8 (4.5–5.3)	4.9 (4.5–5.4); *p* = 0.48	4.9 (4.5–5.2); *p* = 0.16	4.9 (4.6–5.3); *p* = 0.43
HCO₃⁻ (mmol/l)	19 (18.6–20.4)	18.4 (18.0-19.6); *p* = 0.01	18.4 (17.5–19.1); *p* = 0.03	17.8 (16.8–18.4); *p* = 0.006
Temperature (°C)	37.0	37.0	37.0	37.0
Inflammatory Response				
IL-6 (ng/l)	3 (2–4)	1416 (1226–1778); *p* = 0.002	601 (464–672); *p* = 0.002	1212 (1068–1427); *p* = 0.002
Hemolysis index	38 (34–50)	74 (61–95); *p* = 0.008	97 (67–118); *p* = 0.004	109 (76–117); *p* = 0.009

### ODC Parameters

**Fig. 2:**
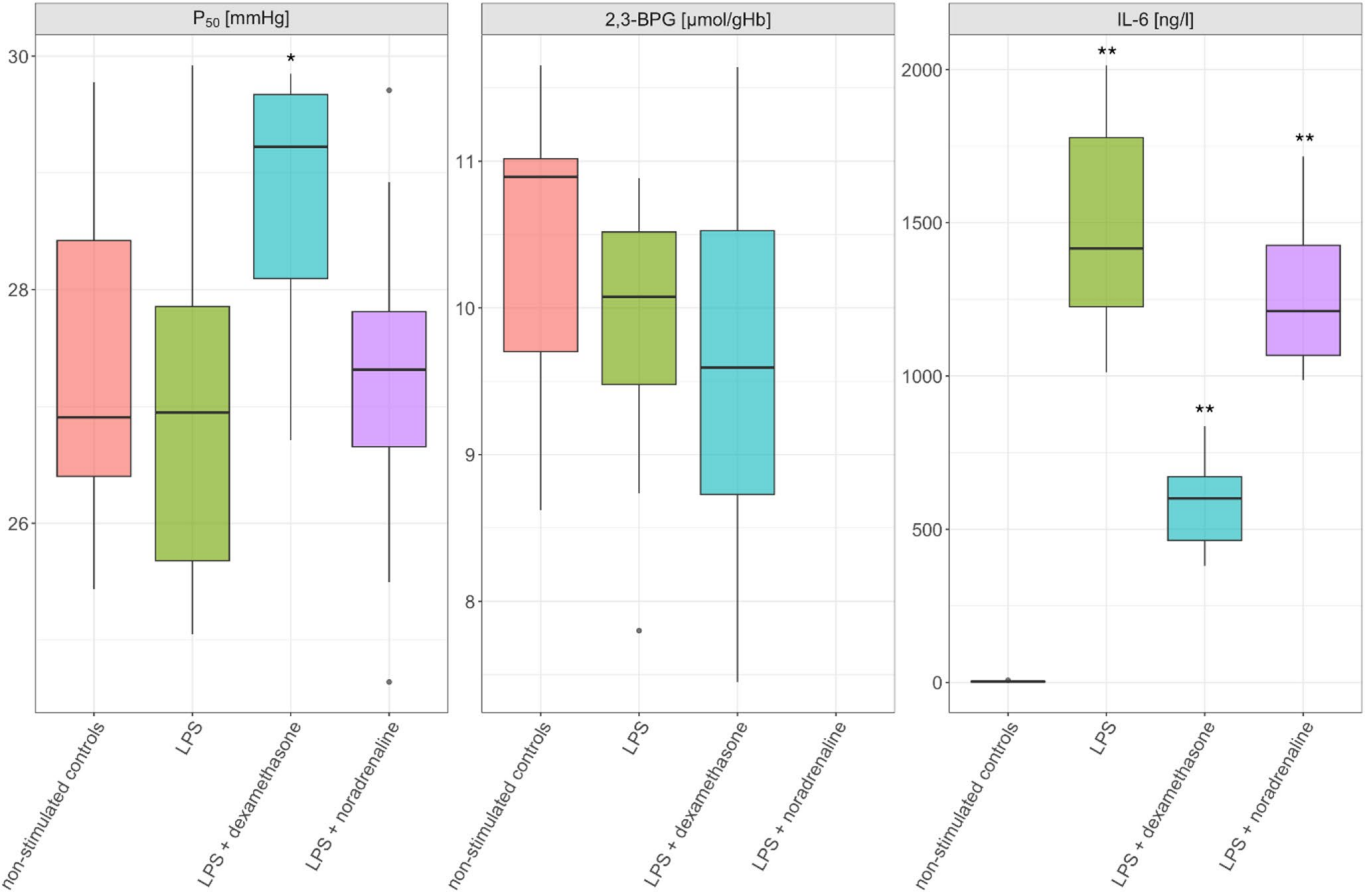
p50, 2,3-BPG and IL-6 values across experimental groups. Data shown as median (Q1-Q3), *n* = 10. *p < 0.05 compared to control group using paired Wilcoxon signed-rank test. 2,3-BPG values for the LPS + noradrenaline group were not analyzed due to resource constraints

ODC parameters remained unchanged following LPS stimulation. p50 values and Hill coefficients showed no significant differences between LPS-stimulated samples and controls (Table 1; Fig. 2). 2,3-BPG concentrations trended lower in the LPS-treated group without reaching statistical significance, whereas ATP concentrations were unaffected by LPS exposure.

### Inflammatory Response

LPS effectively induced inflammatory activation, as evidenced by significantly elevated IL-6 levels compared to control samples. Despite this robust inflammatory response, the primary oxygen-binding characteristics of hemoglobin remained stable.

### Blood Gas Analysis

Blood gas parameters showed subtle but significant changes following LPS stimulation. HCO₃⁻ concentrations were significantly lower in LPS-treated groups compared to controls, while pH remained unchanged, indicating effective buffering. Additionally, hemolysis indices were significantly higher in LPS-stimulated samples, though values remained within acceptable analytical ranges.

### Effects of Dexamethasone and Noradrenaline

Samples co-incubated with LPS and dexamethasone exhibited significantly higher p50 values compared to LPS alone (29.2 (28.0-29.8) vs. 26.9 (25.4–28.0) mmHg, *p* = 0.03) and Hill coefficients (2.47 (2.32–2.56) vs. 2.65 (2.57–2.86), *p* = 0.002). 2,3-BPG concentrations in the LPS plus dexamethasone group showed a tendency toward lower values compared to LPS alone but did not reach statistical significance. ATP levels were unchanged between LPS-treated samples and those co-incubated with dexamethasone.

Both pharmacological interventions demonstrated significant anti-inflammatory effects. Dexamethasone co-treatment markedly suppressed LPS-induced IL-6 levels by 58% (601 (464–672) vs. 1416 (1226–1778) ng/l, *p* = 0.002), while noradrenaline showed a more modest but significant 14% reduction (1212 (1068–1427) vs. 1416 (1226–1778) ng/l, *p* = 0.013) compared to LPS alone. Noradrenaline treatment neither altered P50 values nor Hill coefficients significantly compared to LPS stimulation alone.

## Discussion

Acute LPS-induced inflammation has no direct effect on the affinity of hemoglobin for oxygen. Neither the p50 value nor the Hill coefficient were significantly altered in erythrocytes exposed to LPS compared to control samples, despite substantial inflammatory activation, as evidenced by elevated IL-6 levels. Interestingly, we observed that dexamethasone pretreatment induced a significant right shift of the ODC in LPS-stimulated samples, suggesting a potential therapeutic mechanism that warrants further investigation.

The lack of significant changes in oxygen binding properties following LPS exposure contradicts our initial hypothesis that inflammation would alter the ODC. This finding is noteworthy given the well-established role of erythrocytes in inflammatory responses and the critical importance of optimal oxygen delivery in septic patients.

While erythrocytes can interact with inflammatory mediators through receptors such as DARC [9, 10], our findings suggest that these interactions may not directly influence the functional oxygen-binding properties of hemoglobin. The core mechanisms determining hemoglobin-oxygen affinity appear to remain stable despite the inflammatory environment, at least in the timeframe and conditions of our experiments.

Conflicting findings exist in the literature regarding 2,3-BPG levels in critically ill patients. These studies were conducted in distinctly heterogeneous populations, making direct comparisons challenging. Studies have reported lower 2,3-BPG levels associated with acidemia in mixed ICU populations but observed no change in the in vivo p50 values [17]. An earlier clinical investigation of nine septic patients demonstrated that the left-shifted oxyhemoglobin curve and diminished 2,3-BPG levels observed in seven of these patients could be reversed by correcting acidosis and hypophosphatemia, suggesting that sepsis itself does not directly alter the ODC [18]. Rather, the altered oxygen-binding characteristics appeared to be secondary consequences of specific metabolic derangements common in critical illness, including acid-base disturbances, phosphate deficiency, and transfusion of stored blood with depleted 2,3-BPG. In our controlled experimental model, intraerythrocytic 2,3-BPG concentrations were numerically lower in LPS-stimulated groups, though not statistically significant. While this reduction would theoretically decrease p50 values, the magnitude was likely too modest to significantly affect oxygen-binding properties. ATP concentrations, as well as pH, remained stable following LPS exposure. This stability in the primary physiological modulators of hemoglobin-oxygen affinity likely explains our observation of unchanged p50 values despite confirmed inflammatory activation, though it is important to note these findings were obtained in an in vitro system that may not replicate the complex in vivo environment.

Although it was not our primary research question, we found it interesting that dexamethasone pretreatment caused a significant right shift of the ODC in LPS-stimulated samples. This shift indicates decreased hemoglobin-oxygen affinity, which could theoretically enhance oxygen release to tissues under inflammatory conditions.

Our finding of increased p50 values despite numerically lower 2,3-BPG levels suggests that dexamethasone influences oxygen binding through mechanisms independent of classical allosteric modulators. Since mature erythrocytes are anucleate and lack DNA, this mechanism must occur through non-genomic pathways. One potential explanation involves dexamethasone’s reported effect on insulin binding to erythrocyte receptors, which could theoretically modify the phosphorylation status of membrane proteins, particularly band 3 [19]. Band 3 serves as a hemoglobin binding site and regulates membrane-cytosolic protein interactions, and its insulin-dependent phosphorylation has been shown to influence binding of both glycolytic enzymes and hemoglobin [20–22]. We hypothesize that dexamethasone alters the ODC through membrane protein modifications rather than changes in allosteric effectors. However, this proposed mechanism requires further investigation to establish a definitive causal relationship.

The right shift of the ODC induced by dexamethasone presents complex clinical implications that warrant careful consideration in critical care settings. While previous studies have documented that prednisone and methylprednisolone can increase erythrocyte 2,3-BPG concentrations, our findings with dexamethasone appear to be novel, as to our knowledge, no prior data exist specifically addressing dexamethasone’s effect on oxygen binding properties and the ODC [23, 24].

The debate regarding whether a leftward or rightward shift of the ODC is beneficial remains unresolved and is highly context-dependent, varying with the underlying pathophysiology [25]. Specifically in sepsis, the optimal ODC position likely depends on whether microcirculatory dysfunction or pulmonary impairment predominates. In the context of septic shock with preserved pulmonary function, a rightward shift might be advantageous by facilitating oxygen release to peripheral tissues, potentially mitigating the effects of microvascular dysregulation and diffusion limitations [2]. In this scenario, dexamethasone’s effect on the ODC could complement its anti-inflammatory properties.

However, in critical pulmonary conditions such as acute respiratory distress syndrome (ARDS) or severe SARS-CoV-2 infection, this dexamethasone-induced right shift could be counterproductive. Under these conditions, a leftward shift with increased oxygen affinity would be favorable from a physiological point of view, due to enhanced oxygen uptake in compromised lungs. This consideration becomes particularly relevant in patients receiving lung-protective ventilation strategies with permissive hypercapnia [26]. Under such conditions, arterial PO₂ values often fall on the steep portion of the ODC, where even minor rightward shifts could significantly reduce hemoglobin saturation and compromise oxygen delivery.

We also investigated noradrenaline, the first-line vasopressor in septic shock management. Our findings confirmed the previously described anti-inflammatory effect of noradrenaline, as evidenced by decreased IL-6 levels in co-incubated samples [13]. However, unlike dexamethasone, noradrenaline did not significantly alter the ODC. This contrasts with our previous work demonstrating that noradrenaline decreases oxygen affinity through a currently unspecified molecular mechanism [5]. This apparent discrepancy may be explained by differences in exposure duration. In our previous study, samples were incubated for only 25 min, whereas the current investigation employed a 2-hour incubation period. Given noradrenaline’s short half-life, it may be speculated that its effects on oxygen binding properties manifest through immediate signaling pathways rather than sustained molecular changes. This time-dependent response may explain why noradrenaline’s impact on the ODC was not detected in our longer-duration experiments, suggesting a temporally distinct mechanism from that of dexamethasone.

Our in vitro approach has inherent limitations in remodeling the complex septic environment. The two-hour LPS exposure cannot capture longer-term effects on erythrocyte function that may develop beyond this timeframe, so our observation is limited to the early phase of inflammation. Furthermore, LPS alone cannot replicate the full inflammatory cascade of clinical sepsis. Nevertheless, our controlled experimental conditions provide valuable insights into direct erythrocyte responses to inflammatory stimuli.

The stability of oxygen-binding properties during inflammatory challenges suggests that sepsis-related tissue hypoxia likely stems primarily from microvascular and mitochondrial dysfunction rather than altered hemoglobin characteristics.

The dexamethasone-induced right shift of the ODC warrants further investigation through dose-response studies. Additional in vivo studies in both human and animal models would be invaluable for complementing our findings in the complex septic environment and determining their clinical significance. Future research should also explore potential delayed effects of chronic inflammation on erythrocyte function that may not be evident in acute models.

## Conclusion

In conclusion, our study demonstrates that acute exposure to LPS does not directly alter the oxygen binding properties of erythrocytes. The observed right shift of the ODC with dexamethasone pretreatment represents a novel finding that may have clinical relevance in ARDS and COVID-19 management. These results contribute to our understanding of oxygen transport during inflammatory conditions and highlight the need for further investigation into the complex interplay between inflammation, erythrocyte function, and tissue oxygenation in septic patients.

## Data Availability

The data that support thefindings of this study are available from the corresponding author upon reasonable request.

## Abbreviations

2,3-BPG: 2,3-bisphosphoglycerate
5-HMF: 5-hydroxymethylfurfural
ARDS: acute respiratory distress syndrome
ATP: Adenosine triphosphate
DARC: Duffy-antigen receptor for chemokines
HCO₃⁻: Bicarbonate
IL-6: Interleukin-6
LPS: Lipopolysaccharide
ODC: Oxygen dissociation curve
pCO₂: Partial pressure of carbon dioxide
pO₂: Partial pressure of oxygen
SARS-CoV-2: Severe acute respiratory syndrome coronavirus 2
SO₂: Oxygen saturation

## Declarations

### Generative AI and AI-assisted technologies in the writing process

Claude Sonnet 4 (Anthropic) was used for formulation assistance in manuscript preparation. After using this tool, the authors reviewed and edited the content as needed and take full responsibility for the content of the publication.
